# Complete Moment Convergence and Mean Convergence for Arrays of Rowwise Extended Negatively Dependent Random Variables

**DOI:** 10.1155/2014/478612

**Published:** 2014-02-05

**Authors:** Yongfeng Wu, Mingzhu Song, Chunhua Wang

**Affiliations:** ^1^College of Mathematics and Computer Science, Tongling University, Tongling 244000, China; ^2^Department of Mathematics and Physics, Anhui Traditional Chinese Medical College, Hefei 230051, China

## Abstract

The authors first present a Rosenthal inequality for sequence of extended negatively dependent (END) random variables. By means of the Rosenthal inequality, the authors obtain some complete moment convergence and mean convergence results for arrays of rowwise END random variables. The results in this paper extend and improve the corresponding theorems by Hu and Taylor (1997).

## 1. Introduction

The concept of the complete convergence was introduced by Hsu and Robbins [1]. A sequence of random variables {*U*_*n*_, *n* ≥ 1} is said to converge completely to a constant *θ* if

(1) $$\begin{array}{c} \sum_{n=1}^{\infty }P\left(\left|U_{n}-\theta \right|>\varepsilon \right)<\infty \;\forall \varepsilon >0. \end{array}$$

In view of the Borel-Cantelli lemma, the above result implies that *U*_*n*_ → *θ* almost surely. Therefore, the complete convergence is a very important tool in establishing almost sure convergence of summation of random variables as well as weighted sums of random variables.

Chow [2] presented the following more general concept of the complete moment convergence. Let {*Z*_*n*_, *n* ≥ 1} be a sequence of random variables and *a*_*n*_ > 0, *b*_*n*_ > 0, and *q* > 0. If

(2) $$\begin{array}{c} \sum_{n=1}^{\infty }a_{n}E{\left\{b_{n}^{-1}\left|Z_{n}\right|-\varepsilon \right\}}_{+}^{q}<\infty \;\text{for}\;\;\text{some}\;\;\text{or}\;\;\text{all}\;\;\varepsilon >0, \end{array}$$

then the above result was called the complete moment convergence.

The following concept of negatively orthant dependent (NOD) random variables was introduced by Ebrahimi and Ghosh [3].

**Definition 1**

The random variables *X*_1_,…, *X*_*k*_ are said to be negatively upper orthant dependent (NUOD) if, for all real *x*_1_,…, *x*_*k*_,

(3) $$\begin{array}{c} P\left(X_{i}>x_{i},i=1,2,\ldots ,k\right)\leq \prod_{i=1}^{k}P\left(X_{i}>x_{i}\right) \end{array}$$

and negatively lower orthant dependent (NLOD) if

(4) $$\begin{array}{c} P\left(X_{i}\leq x_{i},i=1,2,\ldots ,k\right)\leq \prod_{i=1}^{k}P\left(X_{i}\leq x_{i}\right). \end{array}$$

Random variables *X*_1_,…, *X*_*k*_ are said to be NOD if they are both NUOD and NLOD.

Liu [4] extended the above negatively dependent structure and introduced the concept of extended negatively dependent (END) random variables.

**Definition 2**

We call random variables {*X*_*i*_, *i* ≥ 1} END if there exists a constant *M* > 0 such that both

(5) $$\begin{array}{c} P\left(X_{i}\leq x_{i},i=1,2,\ldots ,n\right)\leq M\prod_{i=1}^{n}P\left(X_{i}\leq x_{i}\right),\\ P\left(X_{i}>x_{i},i=1,2,\ldots ,n\right)\leq M\prod_{i=1}^{n}P\left(X_{i}>x_{i}\right) \end{array}$$

hold for each *n* = 1,2,… and all *x*_1_,…, *x*_*n*_.

As described in Liu [4], the END structure is substantially more comprehensive than the NOD structure in that it can reflect not only a negative dependence structure but also a positive one, to some extent. Joag-Dev and Proschan [5] also pointed out that negatively associated (NA) random variables must be NOD and NOD is not necessarily NA. Since NOD implies END, NA random variables are END.

The convergence properties of NOD random sequences were studied in the different aspects. We refer reader to Taylor et al. [6] and Ko et al. [7, 8] for the almost sure convergence; Wu et al. [9] for the weak convergence and *L* ^1^-convergence; Amini and Bozorgnia [10], Gan and Chen [11], Wu [12], Wu and Zhu [13], Qiu et al. [14], and Shen [15] for complete convergence; and Wu and Zhu [13] and Wu et al. [9] for complete moment convergence.

Since the paper of Liu [4] appeared, the probabilistic properties for END random variables have been studied by Chen et al. [16], Wu and Guan [17], and Qiu et al. [18]. Since NOD implies END and a great numbers of articles for NOD random variables have appeared in literature, it is very interesting to investigate convergence properties of this wider END class.

For a triangular array of rowwise independent random variables {*X*_*nk*_, 1 ≤ *k* ≤ *n*, *n* ≥ 1}, we let {*a*_*n*_, *n* ≥ 1} be a sequence of positive real numbers with *a*_*n*_↑*∞*, and {Ψ(*t*)} be a positive, even function such that

(6) $$\begin{array}{c} \frac{\Psi \left(\left|t\right|\right)}{{\left|t\right|}^{p}}↑,\;\;\frac{\Psi \left(\left|t\right|\right)}{{\left|t\right|}^{p+1}}↓\;\text{as}\;\;\left|t\right|↑, \end{array}$$

for some nonnegative integer *p*. Conditions are given as

(7) $$\begin{array}{c} EX_{nk}=0,\;1\leq k\leq n,\;\;n\geq 1, \end{array}$$

(8) $$\begin{array}{c} \sum_{n=1}^{\infty }\sum_{\;k=1}^{n}\frac{E\Psi \left(X_{nk}\right)}{\Psi \left(a_{n}\right)}<\infty , \end{array}$$

(9) $$\begin{array}{c} \sum_{n=1}^{\infty }{\left(\sum_{k=1}^{n}E{\left(\frac{X_{nk}}{a_{n}}\right)}^{2}\right)}^{2k}<\infty , \end{array}$$

where *k* is a positive integer.

Hu and Taylor [19] proved the following theorems.

**Theorem 1 A**

Let {*X*_*nk*_, 1 ≤ *k* ≤ *n*, *n* ≥ 1} be an array of rowwise independent random variables and let {Ψ(*t*)} satisfy (6) for some integer *p* > 2. Then (7), (8), and (9) imply

(10) $$\begin{array}{c} \frac{1}{a_{n}}\sum_{k=1}^{n}X_{nk}⟶0\;almost\;\;surely. \end{array}$$

**Theorem 1 B**

Let {*X*_*nk*_, 1 ≤ *k* ≤ *n*, *n* ≥ 1} be an array of rowwise independent random variables and let {Ψ(*t*)} satisfy (6) for *p* = 1. Then conditions (7) and (8) imply (10).

Sung [20], Gan and Chen [21], and Wu and Zhu [13] extended Theorems A and B to the cases of *B*-valued random elements, NA random variables, and NOD random variables, respectively. The goal of this paper is to study complete moment convergence and mean convergence for arrays of rowwise END random variables.

In this work, the authors first present a Rosenthal inequality for sequence of END random variables. By means of the Rosenthal inequality, the authors obtain the complete moment convergence result for arrays of rowwise END random variables, which extends and improves Theorems A and B. In addition, the authors study mean convergence for arrays of rowwise END random variables which was not considered by Hu and Taylor [19].

Throughout this paper, the symbol *C* represents positive constants whose values may change from one place to another.

## 2. Main Results

**Theorem 3**

Let {*X*_*nk*_, 1 ≤ *k* ≤ *n*, *n* ≥ 1} be an array of rowwise END random variables, and let {*a*_*n*_, *n* ≥ 1} be a sequence of positive real numbers with *a*_*n*_↑*∞*. Also, let {Ψ(*t*)} be a positive, even function satisfying

(11) $$\begin{array}{c} \frac{\Psi \left(\left|t\right|\right)}{{\left|t\right|}^{q}}↑,\;\;\frac{\Psi \left(\left|t\right|\right)}{{\left|t\right|}^{p}}↓\;as\;\;\left|t\right|↑ \end{array}$$

for 1 ≤ *q* < *p*.

(i) If 1 < *p* ≤ 2, then conditions (7) and (8) imply
  (12) $$\begin{array}{c} \sum_{n=1}^{\infty }a_{n}^{-q}E{\left\{\left|\sum_{k=1}^{n}X_{nk}\right|-\varepsilon a_{n}\right\}}_{+}^{q}<\infty \;\forall \varepsilon >0. \end{array}$$
(ii) If *p* > 2, then conditions (7), (8), and
  (13) $$\begin{array}{c} \sum_{n=1}^{\infty }{\left(\sum_{k=1}^{n}\frac{EX_{nk}^{2}I\left(\left|X_{nk}\right|\leq a_{n}\right)}{a_{n}^{2}}\right)}^{s}<\infty \end{array}$$

for *s* > 1 imply (12).

**Theorem 4**

Let {*X*_*nk*_, 1 ≤ *k* ≤ *n*, *n* ≥ 1} be an array of rowwise END random variables, and let {*a*_*n*_, *n* ≥ 1} be a sequence of positive real numbers with *a*_*n*_↑*∞*. Also, let {Ψ(*t*)} be a positive, even function satisfying (11) for 1 ≤ *q* < *p*.

(i) If 1 < *p* ≤ 2, then (7) and
  (14) $$\begin{array}{c} \sum_{k=1}^{n}\frac{E\Psi \left(X_{nk}\right)}{\Psi \left(a_{n}\right)}⟶0\;as\;\;n⟶\infty \end{array}$$
 imply
  (15) $$\begin{array}{c} a_{n}^{-1}\left|\sum_{k=1}^{n}X_{nk}\right|\overset{L^{q}}{⟶}0. \end{array}$$
(ii) If *p* > 2, (7), (14), and
  (16) $$\begin{array}{c} a_{n}^{-2}\sum_{k=1}^{n}EX_{nk}^{2}I\left(\left|X_{nk}\right|\leq a_{n}\right)⟶0\;as\;\;n⟶\infty \end{array}$$
 imply (15).

**Remark 5**

Since an independent random variable sequence is a special END sequence, Theorems 3 and 4 hold for arrays of rowwise independent random variables. Note that

(17) ∑n=1∞an−qE{|∑k=1nXnk|−εan}+q =∑n=1∞an−q∫0∞P(|∑k=1nXnk|>εan+t1/q)dt ≥∑n=1∞an−q∫0(εan)qP(|∑k=1nXnk|>εan+t1/q)dt ≥εq∑n=1∞P(|∑k=1nXnk|>2εan),∑n=1∞P(|∑k=1nXnk|>2εan)<∞

implies (10). Therefore, the conclusion of Theorem 3 is stronger than those of Theorems A and B.

## 3. Proofs

To prove our main results, we need the following lemmas.

**Lemma 6 (see [17])**

Let {*X*_*n*_, *n* ≥ 1} be a sequence of END random variables with mean zero and 0 < *B*_*n*_ = ∑_*k*=1_^*n*^ *EX*_*k*_^2^ < *∞*. Let *S*_*n*_ = ∑_*k*=1_^*n*^ *X*_*k*_; then there exists a constant *M* > 0 such that

(18) P(|Sn|≥x)≤∑k=1nP(|Xk|≥y)+2Mexp⁡(xy−xylog⁡(1+xyBn))

∀*x* > 0 and *y* > 0.

**Lemma 7**

Let {*X*_*n*_, *n* ≥ 1} be a sequence of END random variables with mean zero and *E* | *X*_*k*_|^*p*^ < *∞*, where *k* = 1,2,…, *n* and *p* ≥ 2. Let *S*_*n*_ = ∑_*k*=1_^*n*^ *X*_*k*_; then

(19) $$\begin{array}{c} E{\left|S_{n}\right|}^{p}\leq C\left\{\sum_{k=1}^{n}E{\left|X_{k}\right|}^{p}+{\left(\sum_{k=1}^{n}EX_{k}^{2}\right)}^{p/2}\right\}, \end{array}$$

where *C* is a positive constant depending only on *p*.

**Proof**

Let *B*_*n*_ = ∑_*k*=1_^*n*^ *EX*_*k*_^2^. Noting that

(20) $$\begin{array}{c} E{\left|Y\right|}^{p}=p\int_{0}^{\infty }P\left(\left|Y\right|\geq x\right)x^{p-1}\text{d}x\;\left(E{\left|Y\right|}^{p}<\infty \right), \end{array}$$

by taking *y* = *x*/*p* in (18), we have

(21) E|Sn|p=p∫0∞P(|Sn|≥x)xp−1dx≤p∑k=1n∫0∞P(|Xk|≥xp)xp−1dx +2pep∫0∞(1+x2pBn)−pxp−1dx=pp∑k=1nE|Xk|p+epp1+p/2B(p2,p2)(∑k=1nEXk2)p/2,

where

(22) B(α,β)=∫01xα−1(1−x)β−1dx=∫0∞xα−1(1+x)−(α+β)dx.

Letting *C* = max⁡{*p* ^*p*^, *e* ^*p*^ *p* ^1+*p*/2^ *B*(*p*/2, *p*/2)}, we can get (19) from (21). The proof is complete.

**Lemma 8 (see [4])**

If random variables {*X*_*n*_, *n* ≥ 1} are END, then {*g*_*n*_(*X*_*n*_), *n* ≥ 1} are still END, where {*g*_*n*_(·), *n* ≥ 1} are either all monotone increasing or all monotone decreasing.

**Proof of Theorem 3**

Since

(23) ∑n=1∞an−qE{|∑k=1nXnk|−εan}+q =∑n=1∞an−q∫0∞P{|∑k=1nXnk|−εan>t1/q}dt =∑n=1∞an−q(∫0anqP{|∑k=1nXnk|>εan+t1/q}dt +∫anq∞P{|∑k=1nXnk|>εan+t1/q}dt) ≤∑n=1∞P{|∑k=1nXnk|>εan}  +∑n=1∞an−q∫anq∞P{|∑k=1nXnk|>t1/q}dt =^ I1+I2,

to prove (12), it is enough to prove that *I*_1_ < *∞* and *I*_2_ < *∞*. Note that (11) for *q* ≥ 1 implies

(24) $$\begin{array}{c} \frac{\Psi \left(\left|t\right|\right)}{\left|t\right|}↑,\;\frac{\Psi \left(\left|t\right|\right)}{{\left|t\right|}^{p}}↓\;\text{as}\;\;\left|t\right|↑. \end{array}$$

Following the methods used in the proofs of Theorems 1 and 2 in Gan and Chen [21], we can prove *I*_1_ < *∞*. Here we omit the details of the proofs. To prove (12), it suffices to show *I*_2_ < *∞*. Let

(25) Ynk=−t1/qI(Xnk<−t1/q)+XnkI(|Xnk|≤t1/q)+t1/qI(Xnk>t1/q),Znk=Xnk−Ynk=(Xnk+t1/q)I(Xnk<−t1/q)+(Xnk−t1/q)I(Xnk>t1/q).

It follows from Lemma 8 that {*Y*_*nk*_, 1 ≤ *k* ≤ *n*, *n* ≥ 1} is an array of rowwise END random variables. Obviously

(26) P{|∑k=1nXnk|>t1/q  }≤∑k=1nP{|Xnk|>t1/q}+P{|∑k=1nYnk|>t1/q}.

Hence

(27) I2≤∑n=1∞∑ k=1nan−q∫anq∞P{|Xnk|>t1/q}dt +∑n=1∞an−q∫anq∞P{|∑k=1nYnk|>t1/q}dt=^ I3+I4.

For *I*_3_, we have

(28) I3=∑n=1∞∑ k=1nan−q∫anq∞P{|Xnk|I(|Xnk|>an)>t1/q}dt≤∑n=1∞∑ k=1nan−q∫0∞P{|Xnk|I(|Xnk|>an)>t1/q}dt=∑n=1∞∑ k=1nE|Xnk|qI(|Xnk|>an)anq≤∑n=1∞∑ k=1nEΨ(Xnk)Ψ(an)<∞.

By (11), (7), and (8), we have

(29) max⁡t≥anq t−1/q|∑k=1nEYnk|=max⁡t≥anq t−1/q|∑k=1nEZnk|≤max⁡t≥anq t−1/q∑k=1nE|Xnk|I(|Xnk|>t1/q)≤∑k=1nE|Xnk|qI(|Xnk|>an)anq  ≤∑k=1nEΨ(Xnk)Ψ(an)⟶0.

Therefore, while *n* is sufficiently large,

(30) $$\begin{array}{c} \left|\sum_{k=1}^{n}EY_{nk}\right|\leq \frac{t^{1/q}}{2} \end{array}$$

holds uniformly for *t* ≥ *a*_*n*_^*q*^. Then

(31) $$\begin{array}{c} P\left\{\left|\sum_{k=1}^{n}Y_{nk}\right|>t^{1/q}\right\}\leq P\left\{\left|\sum_{k=1}^{n}\left(Y_{nk}-EY_{nk}\right)\right|>\frac{t^{1/q}}{2}\right\}. \end{array}$$

Then we prove *I*_4_ < *∞*. We firstly consider it for the case (i). Let *d*_*n*_ = [*a*_*n*_] + 1; by (31), Lemma 7, and *C*_*r*_ inequality, we have

(32) I4≤C∑n=1∞∑ k=1nan−q∫anq∞t−2/qEYnk2dt=C∑n=1∞∑ k=1nan−q∫anq∞t−2/qEXnk2I(|Xnk|≤dn)dt +C∑n=1∞∑ k=1nan−q∫anq∞t−2/qEXnk2I(dn<|Xnk|≤t1/q)dt +C∑n=1∞∑ k=1nan−q∫anq∞P{|Xnk|>t1/q}dt=^ I41+I42+I43.

By similar argument as in the proof of *I*_3_ < *∞*, we can get *I*_43_ < *∞*. For *I*_41_, by *q* < 2, (*a*_*n*_ + 1)/*a*_*n*_ → 1 as *n* → *∞*, (11), and (8), we have

(33) I41=C∑n=1∞∑ k=1nan−qEXnk2I(|Xnk|≤dn)∫anq∞t−2/qdt≤C∑n=1∞∑ k=1nEXnk2I(|Xnk|≤dn)an2≤C∑n=1∞∑ k=1n(an+1an)2EXnk2I(|Xnk|≤dn)dn2≤C∑n=1∞∑ k=1nE|Xnk|pI(|Xnk|≤dn)dnp≤C∑n=1∞∑ k=1nEΨ(Xnk)Ψ(dn)≤C∑n=1∞∑ k=1nEΨ(Xnk)Ψ(an)<∞.

For *I*_42_, since

(34) $$\begin{array}{c} C\sum_{n=1}^{\infty }\sum_{k=1}^{n}a_{n}^{-q}\int_{a_{n}^{q}}^{d_{n}^{q}}t^{-2/q}EX_{nk}^{2}I\left(d_{n}<\left|X_{nk}\right|\leq t^{1/q}\right)\text{d}t=0, \end{array}$$

we have

(35) $$\begin{array}{c} I_{42}=C\sum_{n=1}^{\infty }\sum_{\;k=1}^{n}a_{n}^{-q}\int_{d_{n}^{q}}^{\infty }t^{-2/q}EX_{nk}^{2}I\left(d_{n}<\left|X_{nk}\right|\leq t^{1/q}\right)\text{d}t. \end{array}$$

Let *t* = *u* ^*q*^; by 1 ≤ *q* < 2, (11), and (8), we have

(36) I42=C∑n=1∞∑ k=1nan−q∫dn∞uq−3EXnk2I(dn<|Xnk|≤u)du=C∑n=1∞∑ k=1nan−q∑m=dn∞∫mm+1uq−3EXnk2I(dn<|Xnk|≤u)du≤C∑n=1∞∑ k=1nan−q∑m=dn∞mq−3EXnk2I(dn<|Xnk|≤m+1)≤C∑n=1∞∑ k=1nan−q∑m=dn∞mq−3∑s=dnmEXnk2I(s<|Xnk|≤s+1)≤C∑n=1∞∑ k=1nan−q∑s=dn∞EXnk2I(s<|Xnk|≤s+1)∑m=s∞mq−3≤C∑n=1∞∑ k=1nan−q∑s=dn∞sq−2EXnk2I(s<|Xnk|≤s+1)≤C∑n=1∞∑ k=1nan−qE|Xnk|qI(|Xnk|>dn)≤C∑n=1∞∑ k=1nE|Xnk|qI(|Xnk|>an)anq≤C∑n=1∞∑ k=1nEΨ(Xnk)Ψ(an)<∞.

Secondly, we prove *I*_4_ < *∞* for the case (ii). By (31), Markov inequality, Lemma 7, and *C*_*r*_ inequality, we have

(37) I4≤C∑n=1∞an−q∫anq∞t−p/qE|∑k=1n(Ynk−EYnk)|pdt≤C∑n=1∞an−q∫anq∞t−p/q[∑k=1nE|Ynk|p+(∑k=1nEYnk2)p/2]dt≤C∑n=1∞∑k=1nan−q∫anq∞t−p/qE|Ynk|pdt+C∑n=1∞an−q∫anq∞t−p/q(∑k=1nEYnk2)p/2dt =^ I44+I45.

For *I*_44_, we have

(38) I44=C∑n=1∞∑k=1nan−q∫anq∞t−p/qE|Xnk|pI(|Xnk|≤dn)dt +C∑n=1∞∑k=1nan−q∫anq∞t−p/qE|Xnk|pI(dn<|Xnk|≤t1/q)dt +C∑n=1∞∑k=1nan−q∫anq∞P{|Xnk|>t1/q}dt=^ I44′+I44′′+I44′′′.

By similar argument as in the proof of *I*_41_ < *∞* and *I*_42_ < *∞* (replacing exponent 2 into *p*), we can get *I*_44_′ < *∞* and *I*_44_′′ < *∞*. By similar argument as in the proof of *I*_3_ < *∞*, we can get *I*_44_′′′ < *∞*.

For *I*_45_, by *p* > 2, we have

(39) I45=C∑n=1∞an−q∫anq∞t−p/q(∑k=1nEXnk2I(|Xnk|≤an)+∑k=1nEXnk2I(an<|Xnk|≤t1/q)+ t2/q∑k=1nP(|Xnk|>t1/q))p/2dt≤C∑n=1∞an−q∫anq∞t−p/q(∑k=1nEXnk2I(|Xnk|≤an))p/2dt +C∑n=1∞an−q∫anq∞(t−2/q∑k=1nEXnk2 ×I(an<|Xnk|≤t1/q))p/2dt +C∑n=1∞an−q∫anq∞(∑k=1nP(|Xnk|>t1/q))p/2dt=^ I45′+I45′′+I45′′′.

By *p* > *q*, *p* > 2, and (13), we have

(40) I45′=C∑n=1∞an−q(∑k=1nEXnk2I(|Xnk|≤an))p/2∫anq∞t−p/qdt≤C∑n=1∞(∑k=1nEXnk2I(|Xnk|≤an)an2)p/2<∞.

Then we prove *I*_45_′′ < *∞*. To start with, we consider it for the case 1 ≤ *q* ≤ 2. By *p* > 2, (11), and (8), we have

(41) I45′′≤C∑n=1∞an−q∫anq∞(t−1∑k=1nE|Xnk|q  ×I(an<|Xnk|≤t1/q))p/2dt≤C∑n=1∞an−q∫anq∞(t−1∑k=1nE|Xnk|qI(|Xnk|>an))p/2dt=C∑n=1∞an−q(∑k=1nE|Xnk|qI(|Xnk|>an))p/2∫anq∞t−p/2dt≤C∑n=1∞(∑k=1nE|Xnk|qI(|Xnk|>an)anq)p/2≤C(∑n=1∞∑ k=1nEΨ(Xnk)Ψ(an))p/2<∞.

Secondly, we prove *I*_45_′′ < *∞* for the case 2 < *q* < *p*. By (11) and (8), we have

(42) I45′′≤C∑n=1∞an−q∫anq∞(t−2/q∑k=1nEXnk2I(|Xnk|>an))p/2dt=C∑n=1∞an−q(∑k=1nEXnk2I(|Xnk|>an))p/2∫anq∞t−p/qdt≤C∑n=1∞(∑k=1nEXnk2I(|Xnk|>an)an2)p/2        (since  2<q<p)≤C∑n=1∞(∑k=1nE|Xnk|qI(|Xnk|>an)anq)p/2≤C(∑n=1∞∑ k=1nEΨ(Xnk)Ψ(an))p/2<∞.

Finally, we prove *I*_45_′′′ < *∞*. From (11), we know Ψ(|*t*|)↑ as |*t* | ↑. Hence, we have

(43) max⁡t≥anq∑k=1nP(|Xnk|>t1/q)≤∑k=1nP(|Xnk|>an)≤∑k=1nEΨ(|Xnk|)Ψ(an)=∑k=1nEΨ(Xnk)Ψ(an)⟶0 as  n⟶∞.

Therefore, while *n* is sufficiently large,

(44) $$\begin{array}{c} \sum_{k=1}^{n}P\left(\left|X_{nk}\right|>t^{1/q}\right)<1 \end{array}$$

holds uniformly for *t* ≥ *a*_*n*_^*q*^. By (44), *p* > 2, and similar argument as in the proof of *I*_3_ < *∞*, we can get

(45) I45′′′≤C∑n=1∞∑ k=1nan−q∫anq∞P(|Xnk|>t1/q)dt≤C∑n=1∞∑ k=1nEΨ(Xnk)Ψ(an)<∞.

The proof is complete.

**Proof of Theorem 4**

Following the notations of the proof in Theorem 3. To start with, we prove (15) for the case 1 < *p* ≤ 2. For all *ε* > 0,

(46) E(an−1|∑k=1nXnk|)q=an−q∫0∞P(|∑k=1nXnk|>t1/q)dt≤ε+an−q∫anqε∞P(|∑k=1nXnk|>t1/q  )dt≤ε+an−q∫anqε∞∑k=1nP{|Xnk|>t1/q}dt +an−q∫anqε∞P{|∑k=1nYnk|>t1/q}dt =^ ε+I5+I6.

Without loss of generality we may assume 0 < *ε* < 1. By Markov inequality, (11), and (14), we have

(47) I5≤∑k=1nan−q∫anqε∞P{|Xnk|I(anε1/q<|Xnk|≤an)>t1/q}dt +∑k=1nan−q∫anqε∞P{|Xnk|I(|Xnk|>an)>t1/q}dt≤∑k=1nan−qE|Xnk|pI(|Xnk|≤an)∫anqε∞t−p/qdt +∑k=1nan−q∫0∞P{|Xnk|I(|Xnk|>an)>t1/q}dt≤Cε1−p/q∑k=1nE|Xnk|panpI(|Xnk|≤an) +∑k=1nan−qE|Xnk|qI(|Xnk|>an)≤(Cε1−p/q+1)∑k=1nEΨ(Xnk)Ψ(an)⟶0 as  n⟶∞.

From (11), (7), and (14), we have

(48) max⁡t≥anqε t−1/q|∑k=1nEYnk| =max⁡t≥anqε t−1/q|∑k=1nEZnk| ≤an−1ε−1/q∑k=1nE|Xnk|I(|Xnk|>anε1/q) ≤ε−1/q∑k=1nE|Xnk|qanqI(|Xnk|>an)  +ε−p/q∑k=1nE|Xnk|panpI(anε1/q<|Xnk|≤an) ≤(ε−1/q+ε−p/q)∑k=1nEΨ(Xnk)Ψ(an)⟶0 as  n⟶∞.

Therefore, while *n* is sufficiently large, for *t* ≥ *a*_*n*_^*q*^ *ε*, we have (31). Let *d*_*n*_ = [*a*_*n*_] + 1; by (31), Lemma 7, and *C*_*r*_ inequality, we have

(49) I6≤C∑k=1nan−q∫anqε∞t−2/qE(Ynk−EYnk)2dt≤C∑k=1nan−q∫anqε∞t−2/qEYnk2dt=C∑k=1nan−q∫anqε∞t−2/qEXnk2I(|Xnk|≤dn)dt +C∑k=1nan−q∫anqε∞t−2/qEXnk2I(dn<|Xnk|≤t1/q)dt +C∑k=1nan−q∫anqε∞P(|Xnk|>t1/q)dt =^ I7+I8+I9.

By similar argument as in the proof of *I*_41_ < *∞*, we can prove

(50) $$\begin{array}{c} I_{7}\leq C\varepsilon^{1-2/q}\sum_{k=1}^{n}\frac{E\Psi \left(X_{nk}\right)}{\Psi \left(a_{n}\right)}⟶0\;\text{as}\;\;n⟶\infty . \end{array}$$

For *I*_8_, since

(51) $$\begin{array}{c} C\sum_{k=1}^{n}a_{n}^{-q}\int_{a_{n}^{q}\varepsilon }^{d_{n}^{q}}t^{-2/q}EX_{nk}^{2}I\left(d_{n}<\left|X_{nk}\right|\leq t^{1/q}\right)\text{d}t=0, \end{array}$$

we have

(52) $$\begin{array}{c} I_{8}=C\sum_{k=1}^{n}a_{n}^{-q}\int_{d_{n}^{q}}^{\infty }t^{-2/q}EX_{nk}^{2}I\left(d_{n}<\left|X_{nk}\right|\leq t^{1/q}\right)\text{d}t. \end{array}$$

Therefore, by similar argument as in the proof of *I*_42_ < *∞*, we can prove

(53) $$\begin{array}{c} I_{8}\leq C\sum_{k=1}^{n}\frac{E\Psi \left(X_{nk}\right)}{\Psi \left(a_{n}\right)}⟶0\;\text{as}\;\;n⟶\infty . \end{array}$$

By similar argument as in the proof of *I*_5_ → 0, we can prove *I*_9_ → 0.

The proof of (15) for the case *p* > 2 is similar to that of (ii) in Theorem 3, so we omit the details. The proof is complete.
